# European Urban Strategies for Attracting Highly Skilled Migrants

**DOI:** 10.1134/S1019331622080068

**Published:** 2022-06-29

**Authors:** Yu. D. Kvashnin

**Affiliations:** Primakov National Research Institute of World Economy and International Relations, Russian Academy of Sciences, Moscow, Russia

**Keywords:** European Union, global cities, labor market, labor migration, highly skilled migration, migration policy, brain drain

## Abstract

In the context of globalization and rapid technological changes, the preservation of human capital and its multiplication are becoming an increasingly important factor of economic growth. These challenges are particularly acute for the European Union and the UK, which have been gradually losing their competitive positions in the world economy over the past decades. In this context it is particularly important to analyze policies aimed at stimulating highly skilled migration carried out at different levels, including the municipal level. Despite their limited competences in the field of migration regulation, municipal administrations are able to influence its dynamics and structure by creating a comfortable urban environment and housing policy, increasing transport accessibility, facilitating employment of local university graduates, supporting return migration, and using other soft measures to attract highly qualified specialists and representatives of the creative class. This process, however, has both winners and losers. Many cities, primarily in the peripheral EU countries, are not able to cope with the competition and handle the ever increasing brain drain problem, which requires comprehensive solutions involving not only municipal administrations but also central authorities and supranational European institutions.

## PROBLEM STATEMENT

In the modern world, attracting and retaining highly skilled migrants (HSMs)^1^ is an indispensable condition for competitiveness. The “fight for talent” is becoming an increasingly serious challenge, especially for those countries that are faced with the problem of reproducing human capital. For the European Union (EU), this challenge is most relevant due to unfavorable demographic trends: an aging population, an increasing strain on social security systems, a shortage of skilled workers exacerbated by the brain drain from less prosperous countries and regions, and, as a result, a growing backlog in the field of science and technology (Kahanec, Zimmermann, 2010). The situation is complicated by the fact that migration flows to the EU (and to a lesser extent to the UK) are dominated by medium and low-skilled migrants, while HSMs prefer to move to other countries: the United States, Australia, Canada, etc. (*Global Talent Risks* *…,* 2011).

The opinion that the authorities should more actively promote the acceptance and integration of HSMs into the host communities is shared not only by scientists and experts from the EU but also by political parties and movements of various ideological orientations, ranging from left-wing radicals to right-wing populists (Potemkin, 2019). The main differences in their views lie in the specific measures that they propose should be taken and the level at which this should be done.

Currently, the main actors that regulate migration processes are the national governments of the countries participating in the integration association. It is they, as noted by N.N. Bol’shova, “who develop special programs to attract the best talent, which actually equalize the socioeconomic rights of the HSMs with their own citizens: they introduce preferential immigration regimes, simplify the rules for granting residence permits, and open access to national labor markets, as well as social insurance systems” (Bol’shova, 2017).

At the same time, two new trends have become increasingly clear in the EU over the past decades. On the one hand, some of the relevant functions flow from the national level to the supranational. On the other hand, regional and especially municipal authorities strive to use the HSMs as a development resource and implement measures that create the most favorable living and working conditions for them.

The issues of managing highly skilled migration at the level of European institutions are considered in detail both in the foreign and Russian scientific literature, primarily in the context of the introduction of a blue card in the EU, which provides third-country nationals with the right to employment in most EU countries (Burmann et al., 2018; Trofimova, Chetverikova, 2019; Bisson, 2020, and others). Also, an extensive number of works is devoted to national approaches to attracting HSMs (Romanova, 2015; Berkhout et al., 2016; Godovanyuk, 2020).

However, municipal strategies in this area and the specific measures of municipal authorities have been studied rather poorly. Scientific articles on this topic, as a rule, analyze examples of individual cities or compare urban practices within one of the EU countries (most often in Germany and the Netherlands). In this article, the author tries to fill the research gap and discover the opportunities that European cities offer to prevent the brain drain and increase their own attractiveness in the eyes of the HSM, as well as the tools that are most needed to form a policy aimed at achieving this goal.

## MIGRATION MANAGEMENT AT THE CITY LEVEL: LIMITATIONS AND OPPORTUNITIES

The growing role of a highly skilled workforce as a key factor in economic development is a global development vector that is most felt at the municipal level, as cities have become the central nodes of a globalizing economy and, as a result, key participants in the competition for investment and talent. At the same time, the municipal authorities in most European cities are still relatively little involved in attracting and retaining HSMs due to two circumstances.

First, migration processes in general and the migration of highly qualified specialists in particular strongly depend on the trajectory of the previous development (“rut effect”). As a rule, HSMs move to those cities that have already attracted labor and leave settlements that lack sufficient employment opportunities, thereby contributing to a decrease in the competitiveness of the latter. In other words, European cities can become magnets for immigrants due to a whole range of factors: economic conditions, the situation on the labor market, the quality of life, the institutional environment and stability, the geographical location of the city, the presence of a formed diaspora, etc. (*Study on the Movement* …, 2018), in the absence of a targeted policy of municipal administrations to attract highly skilled labor. This trend was especially pronounced in global European cities, which for many years to come secured an influx of HSMs due to their position as international financial centers (London, Frankfurt am Main, Amsterdam), centers of a concentration of high-tech industries and the development of science and innovation (Paris, Vienna), or their administrative significance (Brussels, Strasbourg).

It is obvious that in the presence of undeniable competitive advantages, municipal administrations may not attach much importance to this policy direction. In particular, in Frankfurt, the city strategy is focused on increasing the level of education and competence of the Germans themselves, while attracting HSMs from abroad occupies the lowest priority (Bulatov et al., 2021). Being the second most important international financial center in Europe (after London), the city attracts tens of thousands of highly qualified foreign specialists and does not feel the need to take additional measures. Another vivid example is Limassol (Republic of Cyprus), which in just a few decades has turned from a minor port into the second-most-populated city in the Greek part of the island and the most populated Cypriot city in terms of HSMs. The status of the city has risen as a result of a combination of three circumstances: the Turkish occupation of the former main port of Cyprus, Famagusta; the civil war in Lebanon, as a result of which Beirut lost its role as the financial capital of the Eastern Mediterranean (with the result that the international capital there moved to Cyprus); and the business-friendly national tax policy. Due to the extremely high fiscal centralization by European standards, the municipal authorities had almost no influence on this spontaneous process.^2^

Second, as noted, although the competence of municipal administrations in migration management varies by country, in general, it is significantly inferior to the competence of national governments and pan-European authorities. In fact, municipalities play a supporting role in regulating migration. They are forced to act within a strictly defined legal framework, as a rule, without having the right to initiate legislation,^3^ and quite often in conditions of severe financial constraints (for example, in 2020, in order to support the population, the governments of a number of countries reduced local taxes, which had an extremely negative impact on the municipal budgets). In addition, many of municipalities face the more pressing problem of an influx of low-skilled labor, which forces them to focus on the economic and sociocultural integration of refugees and economic migrants, postponing the involvement of HSMs until better times (Kvashnin, 2020).

There is also another important point to consider: the fight to attract talent is not a mandatory but an optional part of municipal policies, which can therefore be ignored by the administration. In this situation, transitory factors such as the interest of municipal leaders in developing appropriate strategies, the ability to mobilize the financial resources necessary for their implementation, and established contacts with urban entrepreneurs who are looking for talented employees become even more important. The latter is especially important: as “municipalities, being state institutions, cannot formulate migration strategies on their own” and must work closely with companies and business associations (Kühn, 2018).

Despite the limitations listed above, in recent decades, an increasing number of municipal administrations consider it important to retain and attract a highly skilled workforce. This is particularly the case in cities where the preservation of human capital has become an existential problem due to the brain drain, and those that consider the fight to attract talented employees as an additional opportunity to develop based on building a knowledge economy.

The former focus on retaining talent and bringing back migrants who have left the city in search of a better life through a wide range of measures, ranging from informing the HSM of available vacancies to direct financial support for highly skilled workers. This approach is mainly typical for the cities of East Germany after the unification of the country, countries in Central and Eastern Europe, many of which were depopulated due to mass emigration, and Southern Europe, where a similar process unfolded during the debt crisis and recession in the late 2000s and early 2010s. This group also includes university cities in Europe, which attract many students from the EU and third countries, but cannot retain them after graduation.

The second group usually focuses on improving the quality of the urban environment by improving accessibility to public transport, developing their housing infrastructure, promoting cultural diversity and tolerance, etc. In some cases, the approaches complement each other, and the policy of cities in this area becomes complex, covering various groups of HSMs, for each of which a different policy tool is used.

## URBAN PRACTICES FOR ATTRACTING HIGHLY SKILLED MIGRANTS

An analysis of urban practices shows that, despite the seeming limited competence of municipal authorities, they have more opportunities to influence the inflow of HSMs than it seems at first glance. Some of these policies deserve special attention.

**Information support for HSMs.** Informing migrants about the procedure for legalizing their stay and position at work, available vacancies, the specifics of education and healthcare services, and the features of everyday life in the city is perhaps the most common practice that is implemented with the direct participation of municipal administrations. In many European cities there are information centers and special Internet sites for migrants. Special information is posted on social networks, which can be intended both for those who have settled in the city, and for HSMs who are only considering future employment. The effectiveness of this tool largely depends on the coordination of the efforts of migration services and businesses, as well as the latter’s demand for a highly skilled workforce. Interestingly, through information assistance, the city authorities can affect the structure of the inflow of HSMs, depending on the tasks facing the city. Thus, in Aachen, Germany, the relevant services focus on students and graduates of local universities; and in Bonn, on representatives of the scientific community and potential employees of TNCs.^4^

**Elimination of bureaucratic barriers.** Although residence and work permits are issued by the national authorities, the municipal government can expedite the process. In particular, the administration of the city of Cologne in Germany optimized the work of the responsible institutions in such a way that these permits started being processed and issued within four weeks. There are cases when municipal authorities acted as guarantors when migrants received the necessary documents (mostly for HSMs in managerial positions) (Fobker et al., 2014).

**Creation of specialized services to search for talented HSMs**. Such initiatives are most widespread in cities that specialize in high-tech industries and are experiencing a shortage of highly skilled labor. The problem is especially acute in the cities of Northern Europe, whose position as leaders in the IT field is threatened by a shortage of personnel (in 2022 the shortage of specialists in this field is estimated be 70 000 people).^5^

The pioneer in this direction was the capital of Denmark, where in 1994 the Copenhagen Capacity organization was created.^6^ It was founded by three amtas (regional governments, abolished by the administrative reform of 2007) and two municipalities, Copenhagen and Frederiksberg, with the assistance of the national investment agency Invest in Denmark. The organization regularly conducts recruiting events both inside and outside the country, helps foreign businesses open branches and subsidiaries in Greater Copenhagen, provides them with market reviews, and assists them in finding partners.

A similar policy is being pursued in Tampere, Finland’s second-largest urban agglomeration, which has a five-year Strategic Program for attracting international talent and migration, which is characterized by its unprecedented detail for European cities (Raunio, 2019).^7^ Among the specific measures of the municipal authorities, particular importance is given to holding annual talent summits, creating job sites, and advising local companies on questions regarding the employment of foreign citizens. The main focus is on attracting talent to those sectors of the economy where the city already has a strong competitive position. Thus, to support the gaming industry in the city, a special structure, the Tampere Startup Hub, was created: a business incubator that brought together several dozen young companies and foreign experts in the field of game design.^8^

**Retaining talent.** This policy direction is most developed in cities that suffer from brain drain, but have the opportunity to reproduce human capital through their strong universities. Studies show a rather large variation in student retention rates not only in Europe as a whole, but within individual countries.^9^ Municipal administrations are not able to prevent the departure of graduates, but in coordination with the leadership of universities, they can make sure that the education system takes into account as much as possible the demand that has formed in the city for certain vacancies. Note that this experience is not always successful. The authorities of the Finnish city of Oulu began a program to train psychologists at a local university^10^ in the expectation that its graduates would then go on to work in the municipal sector. However, most of the specialists left for other regions or got jobs in the private sector.

Some city administrations are not limited to interacting with universities, supporting young talent who have recently graduated from university and are faced with the choice of where to start their working life. The approaches here are different and strongly depend on the specifics of the city and the funds available to the municipality. In San Sebastian (Basque Country), one of the most expensive cities in Spain, the authorities are focusing on providing housing for young scientists with a PhD degree. As part of this task, a House for Talent was built in the city with preferential prices for living there. In addition, with the participation of municipal authorities, a scholarship program for talent (payments of up to 750 euros) has been introduced, which allows them to come to the city for a period of 15 days to two months. Poorer cities in Europe (Thessaloniki in Greece, Zagorje ob Savi in Slovenia, Nagykanizsa in Hungary, etc.) are also developing talent retention strategies, but these involve mostly low-cost activities aimed at establishing links between young talent and employers (Cavallini et al., 2018).

**Stimulation of reverse migration.** This policy direction is typical for cities that are losing skilled labor. In it, the measures of direct financial assistance to returning entrepreneurs are most effective. The municipal authorities of Warsaw, together with the Higher School of Management and Finance, with the support of European funds in the early 2010s implemented the “Become Your Own Boss” project, aimed at helping emigrants who decide to return to the country and who are interested in starting their own business in the Polish capital. Its target groups are citizens over the age of 45, Poles whose seniority was interrupted due to the birth of a child, and citizens who lost their jobs abroad through no fault of their own. It was possible to apply for participation through the website from outside Poland. The participants in the project, based on the assessment of their business plans and the professional training they completed, received non-repayable financial assistance of up to PLN40 000 for the creation of their own company and an additional PLN1100 per month for its development (Evers et al., 2014). A similar policy, but at the regional level, is being pursued in Umbria, Italy, where returning entrepreneurs can receive 20 000 euros (Cavallini et al., 2018).

**Use of transport connectivity with cities and large centers to attract HSMs**. Migrants are put off by the high prices, lack of developed infrastructure for leisure and recreation, inhospitable attitude towards foreigners, etc., prevalent in many European cities with ample employment opportunities. The beneficiaries of this situation are the nearby towns and communities, which are able to offer HSMs more comfortable living conditions. The most striking example of this dynamic is the pendulum migration to Luxembourg, the richest country in the EU, where before the coronavirus pandemic, 46% of the workforce were people who lived in small towns in Belgium, France, and Germany^11^ (Arlon, Metz, Trier).

**Improvement of the urban environment.** The administrations of European cities, as a rule, have significant competences (but not always financial resources) in creating a friendly urban space for HSMs—recreational infrastructure and developed areas for permanent residence—and promoting a culture of tolerance and cosmopolitanism. These measures help attract all categories of HSMs. However, they are most focused on the so-called creative class (scientists, journalists, writers, PR specialists, engineers, actors, artists) employed in the postindustrial segments of the economy. When choosing a city to move to, HSMs are guided not by the availability of jobs, but by the comfort of the housing available to them, the quality of leisure activities, the absence of a language barrier, the presence of English-language schools, etc. Such measures are often influenced by American urbanist thought, popularized by R. Florida, which is that cities can change the paradigm of their own development (overcome the rut effect) by investing in the human climate and habitat, which are considered indispensable conditions for attracting talent and creating high-tech industries (Florida, 2005).

In Europe, interest in such approaches is growing, but their impact on urban policy should not be exaggerated. As a rule, the urban space is not improved in order to attract HSMs: appropriate measures are intended for all residents of the city. Moreover, some municipalities strictly adhere to the “melting pot” concept, seeking to minimize property and ethnic segregation and prevent the emergence of “good” areas for HSMs and “bad” ones for low-skilled migrants, as such a situation would threaten social stability in the city. The deliberate creation of neighborhoods for the compact residence of expats is generally not characteristic for the EU or the UK; if they do appear, usually they are spontaneous and against the will of the municipal authorities. There are, however, exceptions, and the most striking one is Barcelona, where, with the assistance of local authorities, “transnational gentrification” and the creation of “foreigners only” enclaves are taking place (Cocola-Gant and Lopez-Gay, 2020).

## ***

Concluding the review of the policies of European cities to stimulate highly skilled migration, it should be emphasized once again that their opportunities in this area are limited, as the main prerogatives are concentrated in the hands of the central authorities. At the same time, the activity of municipal administrations in this industry is growing, which generally confirms the opinion about the “local U-turn” in the management of migration processes that has taken place in recent decades, which is expressed by a number of experts (Zapata-Barrero et al., 2017; Bernt, 2019). The number of tools for attracting HSMs at the municipal level is increasing and new approaches for interaction with various actors—national governments, the business community, job seekers and, of course, the migrants themselves—are emerging. In a number of cases, the municipalities’ measures are proactive in nature and are aimed at using HSMs as an additional resource for economic development. However, few European cities can boast of comprehensive strategies in this area. More often than not, action is taken belatedly and taken in the face of a massive brain drain that threatens the city’s competitive position.

## Footnotes

1 The concept of a *highly skilled migrant* does not have a generally accepted definition. In this article, in accordance with a common (but far from the only) classification in the European scientific literature, the author classifies five categories of migrants as HSMs: (1) senior managers and executives, (2) engineers and technicians, (3) scientists and teachers, (4) entrepreneurs, and (5) students (Mahroum, 2001).

2 City of Limassol: Intercultural profile. Intercultural cities: Building the Future on Diversity. https://rm.coe.int/1680482a41.

3 Switzerland, which is not part of the EU, is an exception to this rule, as here individual legislative decisions in the field of migration can be made through referendums at the municipal level.

4 Die offizielle Webseite der Stadt Aachen. https:// www.aachen.de/index.html); Foreigners Authority in Bonn. https://www.auslaenderaemter.de/info/info_foreigners_authority_in_bonn.php.

5 The Nordics are struggling with a tech talent shortage. Emerging Europe. November 21, 2019. https://emerging-europe.com/ news/the-nordics-are-struggling-with-a-tech-talent-shortage.

6 Find the right talent. Copenhagen capacity. https://www.copcap.com/how-we-help/finding-talent.

7 It should be noted that in American, Canadian, and Australian cities (Pittsburgh, Detroit, Baltimore, Vancouver, Toronto, Sydney, and many others), the development of such strategies began much earlier than in Europe, which largely assured them success in attracting a highly skilled workforce.

8 About. Tampere Game Hub. https://tampere.games/about.

9 For example, in the UK, the retention rate for graduates is 51% in Edinburgh and Glasgow (which is considered a very good indicator), while in Norwich and Southampton, less than a third of the students stay on there after completing their studies. See.: Graduate Retention: Best UK Cities at Keeping Students After Graduation. https://www.whatuni.com/advice/news/graduate-retention-best-uk-cities/82588.

10 Psychology Graduates Shun Municipal Sector. Yle.fi. https://yle.fi/news/3-120616.

11 Luxembourg: the little-known Covid-19 hotbed. The New Statesman. March 23, 2020. https://www.newstatesman.com/ world/2020/03/luxembourg-coronavirus-covid-19-cases-testing-crossborder.
